# The effects of induced emotions on environmental preferences and behavior: An experimental study

**DOI:** 10.1371/journal.pone.0258045

**Published:** 2021-09-30

**Authors:** Lisette Ibanez, Sébastien Roussel

**Affiliations:** 1 CEE-M, Univ. Montpellier, CNRS, INRAE, Institut Agro, Montpellier, France; 2 CEE-M, Univ. Montpellier, CNRS, INRAE, Institut Agro, Univ. Paul Valéry Montpellier, Montpellier, France; Universidad de Granada, SPAIN

## Abstract

Communication policies employed by policymakers and non-governmental organizations (NGOs) often appeal to the emotions to persuade people to adopt virtuous behavior. The aim of this paper is to study the impact of induced emotions on pro-environmental behavior (PEB). We design a three-stage laboratory experiment. In the first stage, we determine the level of the subjects’ environmental awareness. In the second stage, subjects read scripts that place them in realistic hypothetical scenarios designed to induce specific emotions. We implement a 2 x 2 in-between design by varying both the valence and social dimension of the four emotional states induced: happiness, sadness, pride and shame. In the third stage, subjects play a modified dictator game in which the recipient is an environmental non-governmental organization (ENGO). We show that the emotional states of subjects can influence PEB. In particular, negative emotions significantly reduce the average individual amount of donations made to ENGOs. We also find that the precise impact of the emotional states is more complex and appears to be dependent on individuals’ characteristics and awareness for environmental issues. For instance, in positive emotional states, men donate significantly less than women. In addition, a high level of environmental awareness increases donations in subjects experiencing shame and decreases their likelihood to donate when feeling pride. Also, we observe behavioral consistency for negative emotions and rather compensatory behavior for positive emotions.

## Introduction

Nowadays, communication that appeals to the emotions is a key tool employed by policymakers and non-governmental organizations (NGOs) via advertising campaigns, social media or other communication channels to persuade people to adopt virtuous behavior, and this in various domains [1,2]. In the environmental domain, the idea is to trigger an emotional response, such as empathy or sympathy, that acts as a motivator for people to adopt pro-environmental behavior (PEB) by activating intuitive thinking rather than reasoning [3]. Frey and Maier [4] defined prosocial behaviors as behaviors contributing to the interests of others beyond one’s own self-interest, and PEB correspond to these kinds of behaviors applied in the environmental domain, including activities such as recycling, switching to green energy consumption, volunteering or donation to environmental causes [5]. In most cases, such behaviors are costly which prevent individuals from adopting them [6,7]. An example of the use of emotions in triggering PEB is the 1970s “Crying Indian” television ad broadcasted by the Keep America Beautiful non-profit organization. This famous advertisement was successful in increasing the recycling rate and led to increases in community involvement and an 88% reduction of litter across 38 US states. Since 2001, the non-profit organization ACT Responsible (Advertising Community Together) has annually published “The Good Report”, which is an “unique ranking of the world’s best use of creative communications to promote sustainability and social responsibility to raise awareness of major social and environmental issues”, including deforestation, recycling, water conservation and climate change. ACT claims that, “Advertising is a universal language: images and music are full of emotion and words give us the keys to get involved.”

The rationale behind the use of emotional appeal to drive behavior is that emotions play an important role in decision-making, particularly in relation to social dilemmas [8]. For instance, sadness leads people to greater provision of assistance to a welfare cause [9], anger discourages altruistic action and induces a desire for punishment even if it is costly [10–12], positive feelings promote and reward prosocial behavior, even creating a positive feedback loop [13], and guilt reinforces prosociality [14]. It is also shown that donations increase when a charity uses a positive morally congruent emotion [1] or appeals to the donor’s self-interests [15], and thus activates warm-glow motivations. Moreover, when a charity advertising for donations evokes negative emotions, they generate an increase in prospective donations [16] and stimulate PEB in general, even if these behaviors do not last over time [17].

Economic theory has paid little attention to the way emotions impact decision making. Therefore, we set out to experimentally induce positive and negative feelings that are measured by changes in valence [18].

From the existing literature, it is difficult to draw clear-cut behavioral hypotheses. It appears that both positive and negative emotions may lead to behaviors that vary from anti-sociality to prosociality.

Firstly, concerning positive emotional states, most studies conclude that positive emotions increase altruistic and helpful behavior and favor cooperation [19–21]. People derive happiness merely from opportunities to help and give to others with no expectation of concrete gains [22], and the main action trend that is linked with happiness is “savoring the moment” [23]. These results are in line with the “mood maintenance hypothesis”, which stipulates that people adopt behavior in order to preserve their positive emotional state [23]. However, some other papers do not find a positive relationship between positive mood and generosity [12]. Tan and Forgas [24] even conclude that happiness drives people to selfishness and argue that positive moods recruit more internally focused processing, leading to moral compensation behavior [25].

Secondly, negative emotional states also appear to increase helping behaviors [26], promote fairness [24] and generosity [27], and facilitate the cohesion of social groups [28]. De Hooge [29] argues that the negative feeling of shame “motivates people to undertake actions to restore the damaged self” and thus behave prosocially. However, negative emotional states do not always motivate such prosocial behavior. Guilt, for example, can even lead to antisocial behavior [30], as to focus on oneself can lead an individual to hide or withdraw from others [31,32].

In order to better take into account the diversity of (positive and negative) emotions, we also consider here a social dimension, as one of the motivations for behaving in socially appropriate ways is to gain the social recognition and respect of others. In this paper, in addition to exploring the valence dimension, we distinguish between individual (or basic) emotions, e.g., happiness and sadness, on the one hand, and social (or self-conscious) emotions, e.g., pride and shame, on the other hand. In contrast to individual emotions, social emotions lead people to focus on others and on the way their own behavior affects others’ well- being [33]. Moreover, social ties and norms play an important role in the willingness to behave pro-socially and in the enforcement of PEB and cooperation [34,35]. As a consequence, we might expect social emotions to promote more externally-oriented processing and increase an individual’s willingness to comply with social norms.

In our analysis, we focus on a specific prosocial behavior, that is to say, PEB. Various theoretical frameworks consider PEB to be the outcome of growing environmental knowledge, awareness and concern, in general based on altruistic, warm-glow [36] or other prosocial preferences [37]. These frameworks try to “explain the gap between the possession of environmental knowledge and awareness and exhibiting pro-environmental behavior” [6]. In our study, we choose donations to an environmental non-profit organization, which can be considered as an indirect PEB contrary to the act of recycling or the purchase of solar panels, for instance, which both have a direct impact on the environment. Van Leeuwen and Wiepking [38] argue that we “give to national campaigns because it is a pleasurable experience, which in addition makes us feel good about ourselves and to confirm or create a positive self-image of helpfulness, being a good citizen, an influential person, or a righteous believer”. Nevertheless, an important driver of the willingness to donate to an environmental NGO is one’s emotional involvement, i.e., “the ability to have an emotional reaction when confronted with environmental degradation” [6].

In our experimental study, we distinguish environmental awareness (measured by the new environmental paradigm (NEP) scale [39–41]) from indirect PEB (i.e., a voluntary donation to an environmental NGO [6]), and analyze the interplay between subjects’ emotional states and their observed pro-environmental preferences. We expect that individual’s emotions do impact PEB according to their level of environmental awareness.

The novelty of our experimental design is threefold. First, we use an incentivized modified dictator game in order to elicit PEB. In this modified dictator game, the recipient is an ENGO and any donation above zero implies an intrinsic valuation of giving which we interpret as an adequate proxy for pro-environmental preferences [25]. Second, we elicit the ecological awareness of our subjects and measure it via the NEP scale in order to link subjects’ stated environmental values to their real PEB. Third, we distinguish emotional states not only according to their valence but also to their social dimension. Four different types of emotions are considered: “happiness” and “sadness” (low social dimensions with respectively positive and negative valence), as well as “pride” and “shame” (high social dimension with respectively positive and negative valence).

Our main findings show that the emotional states of subjects influence their monetary donations to ENGOs. However, the impact of a subject’s emotional state on their willingness to donate to an ENGO is more subtle and also appears to be dependent on individual characteristics such as gender, awareness of and attachment to environmental issues. For instance, in positive emotional states, men donate significantly smaller amounts than women. In addition, social emotions influence decision making in highly environmentally aware subjects.

The remainder of this paper is organized as follows. Section 2 outlines our hypotheses and the experimental design of the study. In Section 3, we present our results, and in Section 4 we discuss our findings and conclude.

## Methodology and experimental design

### Hypotheses

In this paper, we focus on two individual emotions, i.e., happiness and sadness and two social emotions, i.e., pride and shame and investigate a set of hypotheses to analyze the impact of these emotions on PEB.

We investigate our first hypothesis which is related to general findings on the potential impact of any manipulation of emotions on decision making [12,42]. From this, we hypothesize that being influenced by any emotional state changes subject’s PEB.

#### Hypothesis 1

Induced emotions affect subjects’ pro-environmental behavior (PEB).

Considering social emotions that are linked to social feedback as opposed to individual emotions, we investigate our second hypothesis which emphasizes the social dimension of emotional states. This hypothesis states that being influenced by social emotions would promote more externally oriented processing in subjects [33]) and thus impact PEB differently than individual emotions.

#### Hypothesis 2

Induced social emotions (i.e., Pride and Shame) affect subjects’ pro-environmental behavior (PEB) differently than induced individual emotions (i.e., Happiness and Sadness).

In addition, by considering that the impact of induced emotions depends on individual characteristics, we investigate our third hypothesis to supplement Hypothesis 1. First, we expect gender differences, as women’s social preferences are found to be more situation-specific and malleable [43]. Second, we suppose that individuals with a high degree of environmental awareness will behave in a more environmentally friendly way than individuals with a low degree of environmental awareness. Indeed, following Aguilar-Luzón et al.’s [44] literature review, empirical evidence shows that people who adhere more strongly to ecocentric beliefs are prone to act in favor of the environment. Consequently, investigating the interplay between emotional states and environmental preferences, we suppose that emotions may impact PEB (if any) according to the level of an individual’s environmental awareness.

#### Hypothesis 3

Induced emotions affect subjects’ pro-environmental behavior (PEB) (if any) according to a) their gender, and b) their level of environmental awareness.

### Experimental design

We design and carry out a laboratory experiment to analyze observed behaviors through a set of treatments in which emotions are induced in the subjects [12,42] and in which subjects have the option to make a monetary donation to an environmental non-governmental organization (ENGO) [45]. We consider a monetary donation to an ENGO as an indirect PEB [6] as an adequate proxy for revealing pro-environmental preferences [25].

#### Experimental strategy

In terms of methodology, we structure our experimental strategy as follows.

First, we measure the stated environmental concern of subjects using the new environmental paradigm (NEP) scale [39–41]. The NEP scale records an individual’s environmental beliefs and interprets the total responses as the subject’s degree of environmental awareness or concern. This scale is considered to be valid and reliable as its measurements correlate highly with real PEB [46]. Our aim is to identify individual environmental awareness levels in order to designate our subjects according to a level of environmental awareness. More precisely, we use the NEP scale to measure an individual’s degree of endorsement (from low to high) of an ecological worldview using a Likert-type 15-item survey with scores for each item, ranging between 1 and 5. We use the French version of the new environmental paradigm (NEP) Scale [47]. Respondents are asked to indicate the extent to which they agree (or disagree) with these 15 items. The answers are then used to develop various statistical measures of environmental concern, either by grouping the items into five three-item categories focused on the limits to growth, anti-anthropocentrism, balance of nature, anti-exemptionalism and the current perception of a major ecological crisis; or, by grouping all the items to get overall and average results. Generally speaking, the higher the score, the higher one’s concern about the environment. As a result, in the subsequent analysis we distinguish subjects with a low level of environmental awareness (*NEP-Low*) from those with a high level of environmental awareness (*NEP-High*).

Second, we implement a 2 x 2 design by varying both the valence and social dimension of the emotional states that are induced in the subjects by placing them in hypothetical situations designed to evoke specific emotions (imagined emotion procedure [48]). In psychology, there are various techniques to induce and measure emotional states through exposure to visual, auditive or brain stimuli such as films [49], pictures [17,45], music [21] or exposure to performance tasks. Other techniques include asking subjects to imagine themselves in fictitious situations, recall past experiences or imagine the future based on past experiences [18,21,50,51]. We opted for an emotion induction technique that combines a script reading procedure and autobiographical recall as we ask subjects to write about the situation by mobilizing previous personal experiences. We use this technique because of the relative ease of use [52] and the reliability of autobiographical recall [18,51]. For example, in Ibanez et al. [45] fear was not correctly induced while using pictures in a slideshow. Subjects correctly reported the negative valence but generally expressed sadness rather than fear. Based on the “shame” scenario (imagined shame) developed by de Hooge et al. [48], we designed five scripts: four scripts to induce the emotions “happiness”, “pride”, “sadness”, and “shame”, and a neutral script, exempt of any emotional triggers, to serve as a benchmark (“control”) (see S1 File). Unlike emotion-evoking ads about global warming [17], our scripts have no link to the environment, in order to avoid any priming effect on PEB. All scripts were tested on a sample of students prior to the experiments to ensure that all scenarios induce the desired affective changes. Overall, we implemented five experimental treatments and randomly assigned subjects to one of the five treatments (between-subjects): two experimental treatments with a positive valence and respectively low and high social dimension, i.e., “happiness” (Treatment T1) and “pride” (Treatment T2), and two experimental treatments with a negative valence and respectively low and high social dimension, i.e., “sadness” (Treatment T3) and “shame” (Treatment T4); a control treatment without any emotional change, i.e., “control” (Treatment TC). During the experiment and in order to control for emotional changes [53,54], the subjects were asked to rate two different scales, i.e., one related to valence and one related to their sensitivity to social feedback, on a 0–99-point scale before and after the script reading. The scale is a conversion of a 9-point Likert scale ranging from 0 (not at all) to 8 (completely). The comparison of these two measures, before and after script reading, allowed us to assess changes in subjects’ emotional states. In addition, subjects were also asked to describe their feeling and thus to pick an adjective corresponding to their current emotional state.

Third, subjects played an incentivized modified dictator game [55,56] in which the recipient is an ENGO and any donation above zero implies intrinsic valuation of giving and reveals pro-environmental preferences [25,45]. A social link between the dictator and the recipient generally increases donations [56,57]. Provided with an endowment of €10, subjects were asked how much of their endowment (an integer between €0 and €10) they wished to give to an ENGO. The subjects had the opportunity to choose between three ENGOs to avoid any anchoring effect and to cover international and national actions: World Wildlife Fund (WWF) (international level; the world’s leading nature conservation organization); Greenpeace (international level; the most talked about ENGO linked to non-violent direct actions); and *Fondation pour la Nature et l’Homme* (national level; a French, non-political organization). A description of each ENGO was made available to the subjects. Subjects were asked to choose the ENGO before the emotion induction in order to avoid any latency effect, i.e., subjects played immediately the modified dictator game after the script reading.

At the end of the experiment, we asked subjects to fill out a survey that contains classical socio-demographic questions.

With regards to payment, we divided each subject’s payment into two envelopes: one with the individual’s earnings (including the participation fee; the other with the individual’s contribution to the chosen ENGO. Payment was made privately and we asked subjects to verify that the amounts in the two envelopes were accurate. We then assured subjects that we would subsequently send the monetary donations to the selected ENGOs.

#### Experimental procedure and subject pool

The experiment was conducted at the Laboratory for Experimental Economics in Montpellier (LEEM)–the Review Board of the LEEM experimental lab approved the study–and consisted of 12 sessions ran in June 2017 and in October 2018. Two-hundred and nine (209) subjects at the University of Montpellier were recruited randomly from the LEEM database following the ORSEE software procedure [58], with written consent obtained from the subjects, provided that they had not previously participated in any dictator-game-type experiment. The experiment was single-blind and implemented using the Python programming framework for experimental economics. The subjects received written instructions (see S2 File). The data were analyzed anonymously.

Of the 209 subjects, there were 38 subjects in the happiness treatment (T1), 43 subjects in the pride treatment (T2), 42 subjects in the sadness treatment (T3), 44 subjects in the shame treatment (T4), and 42 subjects in the control treatment (TC). Sample characteristics are presented in Table 1. We collected data on the subjects’ age (*M_Age_* = 24.36 years, *SD =* 6.07), gender (52.2% men; 47.8% women) and education level (on average 37% of subjects were in a BA or BSc program). One may stress that our sample has a small variability in age, which is frequent in lab experiments where the proportion of students participating is high (82.28% in the entire sample). Note that, as shown in the meta-analysis of Engel [56], the variance of individual explanatory factors in the dictator game across studies is often low. Moreover, we distinguish subjects who had previously participated in experiments (87%) from those who had not. A Kruskall-Wallis equality of population test confirms that our sample is well-balanced across treatments for all socio-economic variables.

**Table 1:** Socio-economic characteristics of the sample (*n* = 209).

*Variable*	*Description*	*Mean*	*Standard Deviation (SD)*	*Min*	*Max*	*Kruskal-Wallis test for equality between treatments (probability)*
***Age***	**Continuous**	24.36	6.07	18	57	0.278
***Gender***	**= 0 if Female;** **= 1 if Male**	0.522	0.501	0	1	0.931
***Education level***	**= 0 if Graduated from High School;** **= 1 if Graduated of a Bachelor degree (BA/BSc);** **= 2 if Graduated of a Master’s degree** **(MA/MSc);** **= 3 if if Graduated of a PhD**	1.11	0.715	0	3	0.683
***Experience***	**= 0 if not participated to any economic experiment in the past;** **= 1 if participated to any economic experiment in the past**	0.871	0.336	0	1	0.630

The experiment lasted about one hour. Payments were made privately at the end of the session with average earnings, including the participation fee, equal to €12.

## Results

### Stated environmental concern

First, we gathered information on subjects’ stated level of environmental concern through the NEP scale presented in Table 2. Average NEP scores were statistically similar across treatments and fluctuated between 3.6 and 3.76. In addition, as stated previously, we construct a binary variable to distinguish subjects with a low level of environmental awareness (*NEP-Low*) from those with a high level of environmental awareness (*NEP-High*). Consequently, we used the classical median split at the 3.8-point limit to separate *NEP-Low* from *NEP-High* subjects, and then to form two distinct groups.

**Table 2:** Scores for pro-environmental motivations and equality of population tests (full sample, by treatment).

	*Number of observations*	*Average value of NEP* *(SD)*	*Average value of NEP-Low* *(SD)*	*Average value of NEP-High* *(SD)*
***Happiness (T1)***	38	3.63 (0.52)	3.34 (0.43)	4.12 (0.16)
***Pride (T2)***	43	3.76 (0.41)	3.5 (0.31)	4.1 (0.23)
***Sadness (T3)***	42	3.60 (0.45)	3.38 (0.34)	4.11 (0.17)
***Shame (T4)***	44	3.66 (0.48)	3.36 (0.30)	4.15 (0.25)
***Control (TC)***	42	3.63 (0.48)	3.37 (0.35)	4.15 (0.23)
***Full sample***	209	3.66 (0.47)	3.39 (0.34)	4.13 (0.21)
***Kruskal-Wallis–equality of population test*** ***(p-value)***		0. 531	0.34	0.895

Significant levels

*** *p*<0.01

** *p*<0.05

* *p*<0.1.

### Emotion induction and assessment

As stated previously, we focus on two dimensions of emotional states: valence (from negative valence as unpleasant feelings to positive valence as pleasant feelings) and social feedback (from low sensitivity to high sensitivity regarding social feedback).

Stated values of the subjects’ emotional states at the beginning of the experiment and the way these values evolve after script reading are provided in Table 3.

**Table 3:** Dynamics of the emotional states (by treatment).

	*Average value of the emotional state at arrival*		*Average mean change of the emotional state after script reading (value after script reading–value at arrival)*	
	Valence (*SD)*	Social feedback (*SD)*	Valence (*SD)*	Social feedback (*SD)*
***Happiness(T1)***	76.66	45.24	6.21	-21.70
	(14.25)	(28.12)	(17.87)	(35.13)
***Pride (T2)***	72.07	48	11.67	17.02
	(20.91)	(32.34)	(23.83)	(29.97)
***Sadness (T3)***	71.33	40.62	-37.48	-3.10
	(17.43)	(25.04)	(31.15)	(33.57)
***Shame (T4)***	73.7	53.36	-41.07	12.98
	(17.03)	(29.83)	(31.51)	(21.43)
***Control (TC)***	74.90	49.12	-4.76	10.48
	(15.83)	(28.58)	(22.30)	(25.81)
***Kruskal-Wallis–equality of population test*** ***(p-value)***	0.777	0.369	0.0001***	0.0001***

Significant levels

*** *p*<0.01

** *p*<0.05

* *p*<0.1.

No significant differences either in valence or sensitivity to social feedback were observed among treatments upon arrival (*p* = 0.777 for valence, *p* = 0.369 for social feedback). On the contrary, emotional states for the different treatments were significantly different after the script reading, with a Kruskal-Wallis test significant at less than 1% (***) for both valence and social feedback sensitivity.

To make sure that emotional states were correctly induced, we checked that both indicators (valence and social influence) evolve as expected after script reading for the different treatments. We show that valence evolves positively for the two positive emotions (happiness (T1) and pride (T2)) and negatively for the two negative emotions (sadness (T3) and shame (T4)); and, that the influence of social feedback evolves positively for the two social emotions (pride (T2) and shame (T4)) and negatively for the two individual emotions (happiness (T1) and sadness (T3)).

Simultaneous comparison of all treatments regarding changes in emotional states using multiple hypothesis testing (MHT) [59] is provided in Table 4 (the simultaneous comparison of all treatments is available upon request.). Results are provided successively for the valence and social feedback dimensions.

**Table 4:** Multiple hypothesis testing (MHT) of changes in emotional states (comparison between the control and emotion treatments and between positive and negative emotions).

**Valence**					
		***Unadjusted (p-value)***	***Adjusted (p-value)***		
***Compared treatments***	**Difference in means**	**Remark 3.1**	**Thm 3.1**	**Bonferroni**	**Holm**
***TC vs T1***	10.97	0.016**	0.045*	0.16	0.048**
***TC vs T2***	16.44	0.002***	0.002***	0.017**	0.007***
***TC vs T3***	32.71	0.0001***	0.0001***	0.0001***	0.003***
***TC vs T4***	36.31	0.0001***	0.0001***	0.0001***	0.002***
***T1 vs T2***	5.46	0.229	0.403	1	0.457
***T3 vs T4***	3.59	0.596	0.596	1	0.596
***Social feedback***					
		***Unadjusted (p-value)***	***Adjusted (p-value)***		
***Compared treatments***	**Difference in means**	**Remark 3.1**	**Thm 3.1**	**Bonferroni**	**Holm**
***TC vs T1***	32.19	0.0001***	0.0001***	0.0001***	0.003***
***TC vs T2***	6.55	0.284	0.528	1	0.854
***TC vs T3***	13.57	0.044*	0.135	0.44	0.176
***TC vs T4***	2.5	0.614	0.614	1	0.614
***T1 vs T2***	38.73	0.0001***	0.0001***	0.0001***	0.003***
***T3 vs T4***	16.07	0.011**	0.052*	0.107	0.064*

Significant levels

*** *p*<0.01

** *p*<0.05

* *p*<0.1.

Comparing mean changes across treatments using MHT, we can observe that emotions appear to have been perfectly induced in regards to the valence dimension. Indeed, mean changes in valence of positive emotions, i.e., happiness (T1) and pride (T2) are significantly positive and higher compared to the control treatment (TC). Inversely, mean changes in valence of negative emotions, i.e., sadness (T3) and shame (T4) are significantly negative and lower compared to the control treatment (TC). Moreover, valence levels of the two positive emotions happiness and pride (T1 vs T2) as well as the two negative emotions sadness and shame (T3 vs T4) are not statistically different. With regards to the social dimension, results are less striking. Indeed, we do not find any significant difference between the control treatment and the two social emotions (TC vs T2 and TC vs T4, respectively). However, subjects report social feedback to be of lesser importance in the happiness (T1) and sadness (T3) treatments than in the control treatment (TC). More importantly, the social dimension is significantly different for the two positive emotions (T1 vs T2) and the two negative emotions (T3 vs T4), which means that individuals judge social feedback to be of higher importance when feeling pride (respectively shame) than when feeling happiness (respectively sadness). Additional information on subjects’ reported adjectives corresponding to their current emotional states are available in S1 Table.

### Emotions and monetary donation

#### Monetary donation descriptive statistics

**Fig 1:**
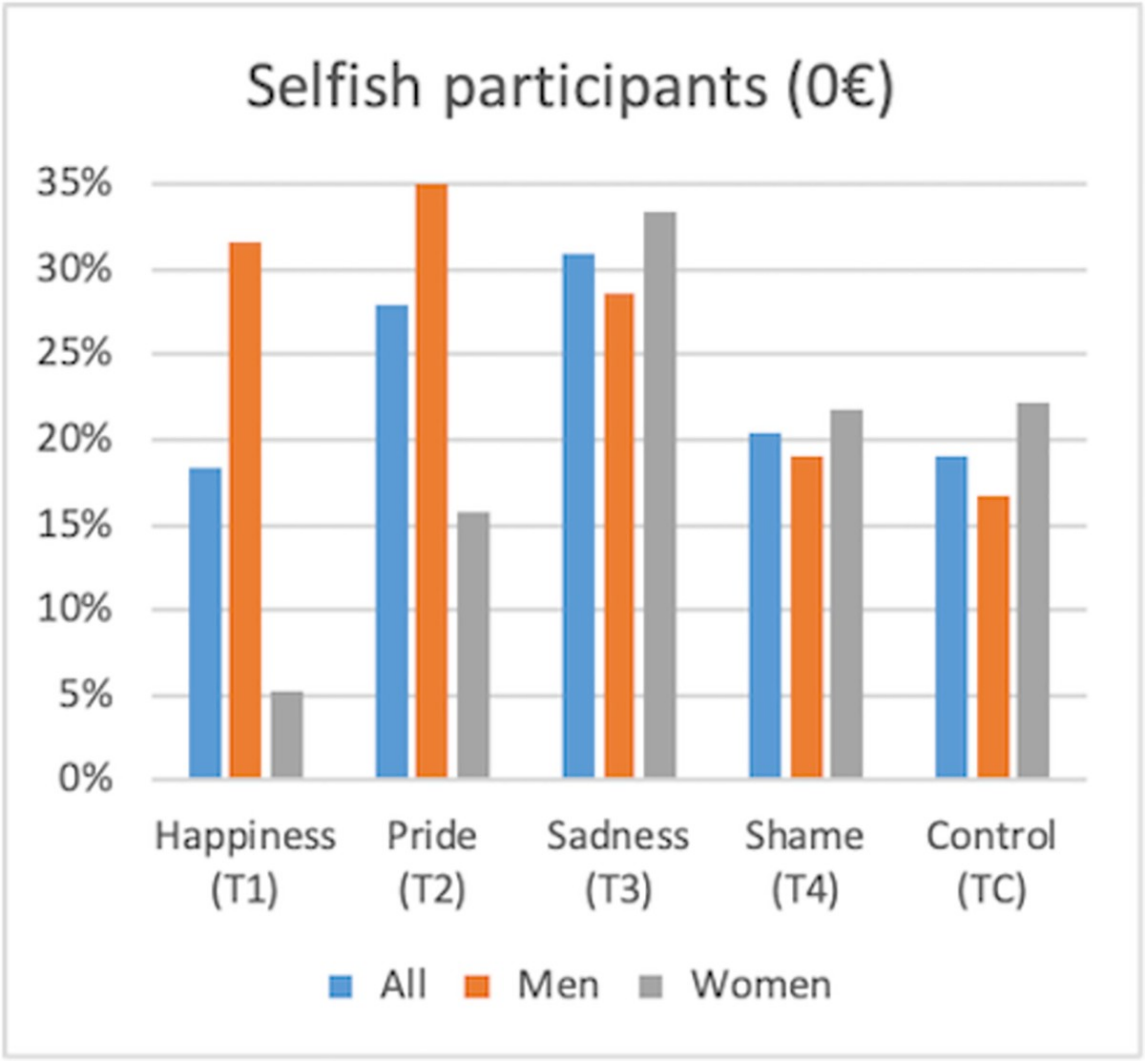
Selfishness and gender effect (by treatment).

**Fig 2:**
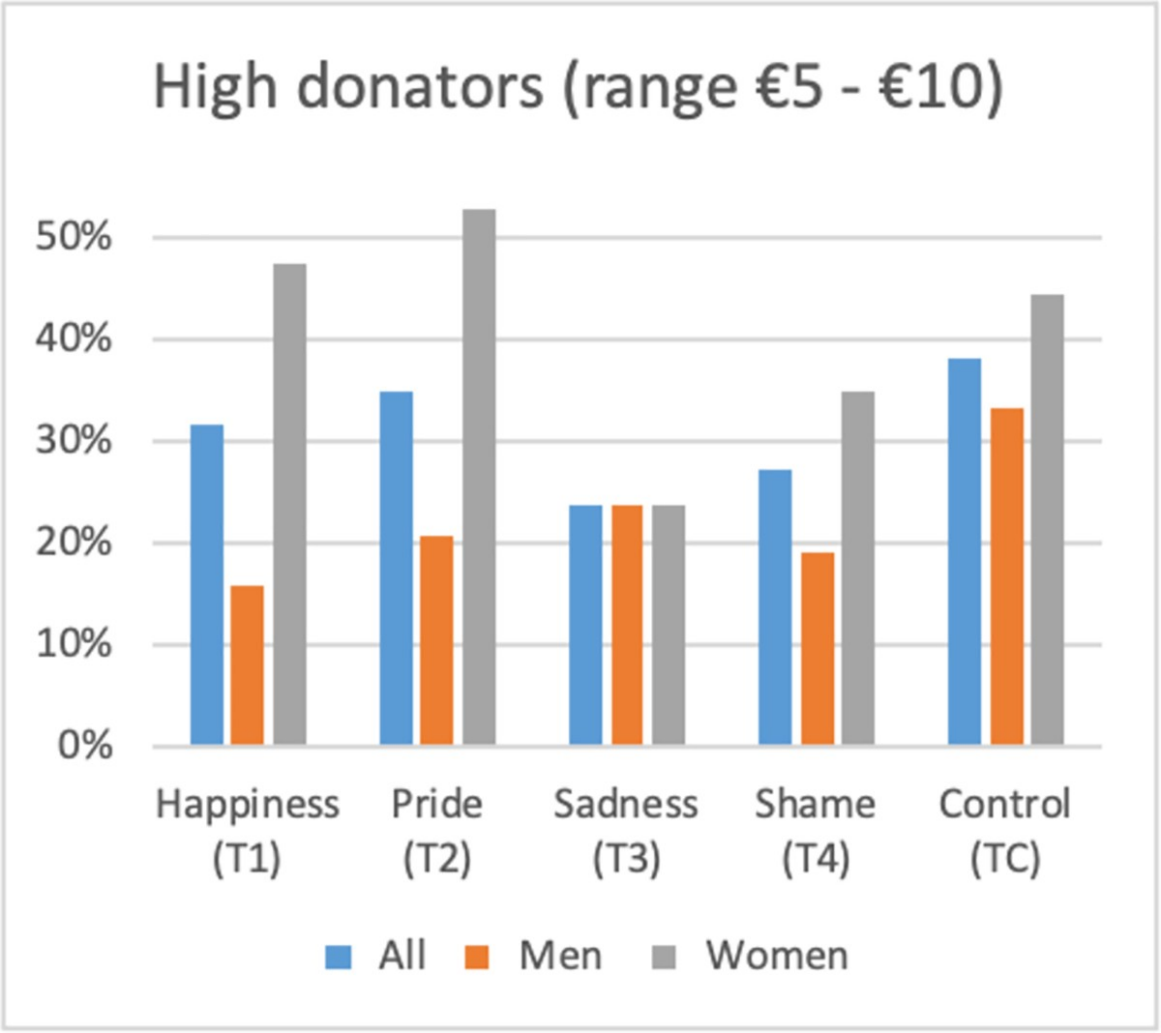
High-level donations and gender effect (by treatment).

Donation decisions are analyzed in Tables 5–7 (regarding Table 6, the simultaneous comparison of all treatments is available upon request) and Figs 1 and 2.

**Table 5:** Donation decisions and gender effect (by treatment and full sample).

	*Sample size*		*Amount given on average (€)* *(SD)*			*Wilcoxon-rank sum test* *(Men-Women)* *(p-value)*
	*N*	% of men	All	Men	Women	
***Happiness (T1)***	38	50%	€3.37 (€3.05)	€2.47 (€3.06)	€4.26 (€2.84)	0.0223**
***Pride*** ***(T2)***	43	55.81%	€3.14 (€3.29)	€2.17 (€2.68)	€4.37 (€3.64)	0.0332**
***Sadness*** ***(T3)***	42	50%	€2.29 (€2.63)	€2 (€2.02)	€2.57 (€3.16)	0.8772
***Shame*** ***(T4)***	44	47.72%	€2.57 (€2.27)	€2.29 (€1.85)	€2.83 (€2.61)	0.7197
***Control*** ***(TC)***	42	57.14%	€3.69 (€3.47)	€3.58 (€3.57)	€3.83 (€3.43)	0.8573
***Full sample***	209	52.15%	€3 (€2.99)	€2.52 (€2.75)	€3.52 (€3.16)	0.0248**
***Kruskal-Wallis test*** ***(p-value)***			0.3519	0.5530	0.2027	

Significant levels

*** *p*<0.01

** *p*<0.05

* *p*<0.1.

**Table 6:** Multiple hypothesis testing (MHT) of average donations (comparison between the control and emotion treatments).

		*Unadjusted (p-value)*	*Adjusted (p-value)*		
*Compared Treatments*	Difference in means	Remark 3.1	Thm 3.1	Bonferroni	Holm
***TC vs T1***	0.3221	0.649	0.649	1	0.649
***TC vs T2***	0.5509	0.4573	0.674	1	0.9147
***TC vs T3***	1.4048	0.0437**	0.1257	0.1747	0.1747
***TC vs T4***	1.1222	0.0793*	0.18	0.3173	0.238

Significant levels

*** *p*<0.01

** *p*<0.05

* *p*<0.1.

**Table 7:** Donation decisions and environmental awareness (by treatment and full sample).

	*NEP-Low* *NEP scale score < 3.8*		*NEP-High* *NEP scale score ≥ 3.8*		*Wilcoxon-rank sum test* *(p-value)*
	*N*	Amount given on average (€) (SD)	*N*	Amount given on average (€) (SD)	
***Happiness*** ***(T1)***	24	€2.917 (€2.903)	14	€4.143 (€3.255)	0.2159
***Pride*** ***(T2)***	24	€2.792 (€2.782)	19	€3.579 (€3.878)	0.9305
***Sadness*** ***(T3)***	29	€2.138 (€2.460)	13	€2.615 (€3.069)	0.6966
***Shame*** ***(T4)***	27	€2.074 (€1.94)	17	€3.353 (€2.573)	0.0928*
***Control*** ***(TC)***	28	€3.214 (€3.51)	14	€4.643 (€3.191)	0.1000*
***Full sample***	132	€2.614 (€2.762)	77	€3.662 (€3.251)	0.0235**
***Kruskal-Wallis test*** ***(p-value)***		0.762		0.449	

Significant levels

*** *p*<0.01

** *p*<0.05

* *p*<0.1.

First of all, the average donation for the entire sample equals €3 (Table 5), and is similar to the results established in the literature for modified dictator games where the recipient is an NGO [56,57].

Where individuals are exposed to emotional states, donations decrease compared to the control treatment group (see S1 Fig). However, these findings are only statistically significant for the two negative emotions, sadness (T3) and shame (T4) at respectively the 5% and 10% levels (Tables 5 and 6 with adjusted values). On average, donations are reduced by more than €1 for people experiencing sadness (-€1.41) or shame (-€1.12). This result is mainly due to the decrease of the number of high-level donators, i.e., subjects who gave to the ENGO an amount within the range of €5 and €10.

With regards to positive emotions, we observe a gender effect (Table 5 and S2 Fig): women donate significantly more than men (respectively €4.26 and €2.47 in the happiness treatment group (T1), and €4.37 and €2.17 in the pride treatment group (T2); statistically significant at the 5% level). This disparity is mainly due to the proportion of selfish participants (i.e., subjects keeping the €10 endowment for themselves) and high donators (subjects giving to the ENGO an amount within the range of €5 and €10) within the different treatments (Figs 1 and 2). When subjects are exposed to positive emotions, men adopt more selfish behavior (32% of the sample in the happiness treatment group (T1) and 38% in the pride treatment group (T2) compared to only 17% of the sample in the control treatment group (TC)) whereas women adopt less selfish behavior (5% of the sample in the happiness treatment group (T1) and 16% in the pride treatment group (T2) compared to only 22% of the sample in the control treatment group (TC)). At the same time, women represent a larger proportion of high donators in the positive emotions treatments (T1 and T2) than in the control treatment group (TC). Indeed 47% of women feeling happiness (T1) and 53% feeling pride (T2) donate an amount above €5 to the ENGO compared to only 44% in the control treatment group (TC). Moreover, only 16% of men feeling happiness (T1) and 21% feeling pride (T2) donate an amount above €5 compared to 33% in the control treatment group (TC). Consequently, we observe more variability in female behavior according to the emotional exposure than in male behavior, which can be explained by the fact that in general women are more sensitive to the protocol and context [60].

Another observation can be made by comparing the environmental awareness of subjects and their average donations to the ENGO (Table 7 and S3 Fig). Average donations by subjects who are highly aware of environmental issues were higher than those less aware of those issues (€3.67 vs €2.61; *p* = 0.0235). This confirms that stated environmental preferences measured by the NEP-scale correlate with observed PEB. This tendency is only statistically confirmed for the control treatment group (TC) and the shame treatment group (T4).

#### Econometric analysis

To carry out a deeper analysis of the factors leading to monetary donations to ENGOs and the role played by the emotional states, we use a set of econometric models (Tables 8.1 and 8.2). Firstly, we perform a censored regression model (Tobit model) to explore the determinants of donation behavior with regards to our choice space. Secondly, to supplement the analysis we use the two-stage Cragg’s [61] hurdle model procedure to further explore the impact of emotions on monetary donation. This procedure combines a participation regression model (Hurdle 0/1 –i.e., a Probit model) to assess the intensive margin of donating (likelihood), with a truncated regression (Hurdle +) to assess the extensive margin of donating (level of donation), conditional to being a donor [62–64]. In other words, we disentangle the participation and quantity dimensions in the monetary donation process within this procedure. To complete the analysis, we compute the marginal effects in order to address the effective monetary impacts as conditional mean estimates from explanatory variables used in both stages of the Cragg-Hurdle model (Tables 9.1 and 9.2).

**Table 8.1:** Treatment effects, intensive and extensive margins of monetary donation/separate emotional states.

Variables	Tobit I	Cragg-Hurdle I		Tobit II	Cragg-Hurdle II	
		Hurdle 0/1 *Likelihood*	Hurdle + *Regression*		Hurdle 0/1 *Likelihood*	Hurdle + *Regression*
***Happiness (T1)***	-0.404	-0.020	-1.161	1.305	0.947	-1.054
	(0.794)	(0.331)	(1.235)	(1.358)	(0.625)	(2.115)
***Pride (T2)***	-0.841	-0.259	-0.687	1.940	1.348*	-0.937
	(0.777)	(0.314)	(1.202)	(1.431)	(0.688)	(2.206)
***Sadness (T3)***	-1.701**	-0.379	-2.245*	-0.484	0.047	-1.812
	(0.783)	(0.311)	(1.353)	(1.363)	(0.534)	(2.327)
***Shame (T4)***	-1.645**	-0.081	-3.474***	-1.074	0.052	-3.500*
	(0.782)	(0.328)	(1.344)	(1.351)	(0.547)	(2.330)
***Control (TC)***	*Ref*.	*Ref*.	*Ref*.	*Ref*.	*Ref*.	*Ref*.
***Age***	-0.011	0.010	-0.057	-0.016	0.014	-0.080
	(0.041)	(0.018)	(0.066)	(0.042)	(0.019)	(0.066)
***Gender (Male)***	-1.016**	-0.219	-1.549*	0.861	0.381	-0.161
	(0.501)	(0.201)	(0.850)	(1.164)	(0.503)	(1.781)
***Education level***	0.014	0.283**	-1.392**	-0.033	0.275*	-1.323**
	(0.356)	(0.144)	(0.661)	(0.356)	(0.153)	(0.649)
***Experience***	-1.982***	-0.712*	-1.857*	-2.117***	-0.752*	-2.023*
	(0.742)	(0.380)	(1.079)	(0.739)	(0.396)	(1.077)
***NEP-High***	1.090**	0.078	2.156**	1.741	0.813	0.590
	(0.515)	(0.209)	(0.861)	(1.206)	(0.598)	(1.775)
***Happiness (T1)*** * ***Gender***				-3.278**	-1.465*	-1.730
				(1.634)	(0.751)	(2.562)
***Pride (T2)*** * ***Gender***				-3.654**	-1.366*	-2.581
				(1.601)	(0.714)	(2.529)
***Sadness (T3)*** * ***Gender***				-1.442	-0.321	-1.948
				(1.605)	(0.654)	(2.754)
***Shame (T4)*** * ***Gender***				-1.115	-0.188	-1.452
				(1.568)	(0.677)	(2.591)
***Happiness (T1)*** * ***NEP-High***				0.058	-0.378	1.877
				(1.684)	(0.811)	(2.510)
***Pride (T2)*** * ***NEP-High***				-1.879	-1.882**	3.255
				(1.662)	(0.777)	(2.513)
***Sadness (T3)*** * ***NEP-High***				-1.037	-0.796	1.339
				(1.692)	(0.744)	(2.783)
***Shame (T4)*** * ***NEP-High***				0.180	-0.083	1.792
				(1.629)	(0.787)	(2.596)
***Constant***	5.535***	1.082*	8.045***	4.548***	0.474	8.707***
	(1.461)	(0.652)	(2.319)	(1.647)	(0.740)	(2.643)
***lnsigma*, *Constant***		1.293*** (0.114) 3.643 (0.414)			1.269***	
					(0.111)	
***/sigma***	3.459			3.397	3.559	
	(0.204)			(0.203)	(0.395)	
**LL**	-471.423	-462.401		-466.923	-452.461	
**LR Chi** ^**2**^ **(9)**	22.89***	38.61***		- 31.90** 0.033	- 58.49*** 0.061	
**LR Chi** ^**2**^ **(17)**	-	-				
**Pseudo R** ^**2**^	0.024	0.040				
**Observations**	209	209		209	209	

Standard errors in parentheses

*** p<0.01

** p<0.05

* p<0.1.

**Table 8.2:** Treatment effects, intensive and extensive margins of monetary donation/individual and social emotions.

Variables	Tobit III	Cragg-Hurdle III	
		Hurdle 0/1 *Likelihood*	Hurdle + *Regression*
***Individual emotions (T1+T3)***	-1.077	-0.218	-1.736
	(0.686)	(0.279)	(1.142)
***Social emotions (T2+T4)***	-1.247*	-0.178	-2.131*
	(0.683)	(0.281)	(1.129)
***Control (TC)***	*Ref*.	*Ref*.	*Ref*.
***Age***	-0.007	0.009	-0.051
	(0.042)	(0.017)	(0.067)
***Gender (Male)***	-1.009**	-0.215	-1.649*
	(0.506)	(0.200)	(0.893)
***Education level***	0.010	0.282**	-1.466**
	(0.359)	(0.143)	(0.688)
***Experience***	-1.910**	-0.766**	-1.624
	(0.742)	(0.380)	(1.114)
***NEP-High***	1.134**	0.079	2.187**
	(0.520)	(0.207)	(0.904)
***Constant***	5.364***	1.144*	7.713***
	(1.466)	(0.654)	(2.419)
***lnsigma*, *Constant***	-	1.324*** (0.117)	
***/sigma***	3.496	3.759	
	(0.206)	(0.441)	
**LL**	-473.259	-465.807	
**LR Chi** ^**2**^ **(7)**	19.23***	31.80***	
**Pseudo R** ^**2**^	0.02	0.033	
**Observations**	209	209	

Standard errors in parentheses

*** p<0.01

** p<0.05

* p<0.1.

**Table 9.1:** Cragg-Hurdle model I–conditional mean estimates (marginal effects) as effective monetary impacts (€)/specific emotional states.

Variables	Margins
***Happiness (T1) versus Control (TC) (Ref*.*)***	-0.562
	(0.682)
***Pride (T2) versus Control (TC) (Ref*.*)***	-0.646
	(0.680)
***Sadness (T3) versus Control (TC) (Ref*.*)***	-1.368**
	(0.646)
***Shame (T4) versus Control (TC) (Ref*.*)***	-1.455**
	(0.612)
***Gender (Male)***	-0.847**
	(0.392)
***Education level***	-0.228
	(0.299)
***Experience***	-1.515***
	(0.579)
***NEP-High***	0.926**
	(0.399)

Standard errors in parentheses; Significant levels

*** p<0.01

** p<0.05

* p<0.1.

**Table 9.2:** Cragg-Hurdle model III–conditional mean estimates (marginal effects) as effective monetary impacts (€)/individual and social emotions.

Variables	Margins
***Individual emotions (T1+T3) versus Control (TC) (Ref*.*)***	-1.077
	(0.686)
***Social emotions (T2+T4) versus Control (TC) (Ref*.*)***	-1.247*
	(0 .683)
***Gender (Male)***	-1.009**
	(0.506)
***Education level***	0.010
	(0359)
***Experience***	-1.910***
	(0.741)
***NEP-High***	1.134**
	(0.520)

Standard errors in parentheses; Significant levels

*** p<0.01

** p<0.05

* p<0.1.

A censored regression model (Tobit model, with 49 left-censored observations) allows us to explore the determinants of donation behavior as a whole. A first model (Tobit I; Table 8.1) investigates whether emotions directly impact donation levels. We observe that the feelings of sadness and shame reduce average donations to the ENGO. However, positive emotions do not significantly modify donation behavior. Interestingly, subjects with a high level of environmental awareness donate more than less environmentally aware subjects. Moreover, men as well as subjects who have already participated in an economic experiment in the past are less generous towards the ENGO. Considering a second model with interaction terms (Tobit II; Table 8.1), negative emotions no longer impact donation levels. Nevertheless, positive emotions influence donation behavior according to gender, i.e., men donate significantly less when experiencing positive emotions than women do. Indeed, women are in general more generous, especially when the stakes are low (e.g., cost of donating, risk of punishment) [65]. However, this does not appear to be true in negative emotional states. A third model (Tobit III; Table 8.2) shows that social emotions negatively impact average donations, and appear to be driven by the impact of shame. This result is somewhat surprising as social emotions, as opposed to individual ones, tend to lead people to focus on the way their behavior affects others’ well-being [33]. Thus, we would have expected social emotions to play an important role in the enforcement of environmental behavior and cooperation [34,35].

The two-stage hurdle model allows us to disentangle the willingness to donate from the amount donated.

In a first model (Cragg-Hurdle I; Table 8.1), we observe that negative emotions impact only the average amount donated but do not influence the probability of donating (or being selfish). Only education level and the fact that subjects have already participated in an economic laboratory experiment decrease the probability of donating. Again, we observe that the average amount donated by men is smaller than the average amount donated by women, when depicting conditional means estimated through marginal effects as effective monetary impact (about €1 less; Table 9.1). Subjects with a high of level environmental awareness who donate, generally donate a higher amount than those with a low level of environmental awareness (about €1 more; Table 9.1). Even if highly educated subjects are keener to donate, the amount donated is lower than that given by less educated subjects. The effective monetary impact of the education level is not significant (Table 9.1). In a second model, when considering interaction terms (Cragg-Hurdle II; Table 8.1), social emotions become determinant for PEB. When a subject feels pride, the probability of actually donating increases, whereas the average amount donated decreases for subjects experiencing shame. One of the explanations of this result might be the “cognitive dissonance” hypothesis, which stipulates that people stick to behavior they are familiar with [66]. Thus, when feeling pride, people adopt behavior to preserve their positive emotional state, and shame motivates anti-social behavior [30]. A third model (Cragg-Hurdle III model; Table 8.2) confirms the negative impact of social emotions on the average amount donated, which represents an effective monetary reduction in donations of €1.25 (Table 9.2), the decrease being driven by shame.

In addition, we can also observe that the decrease of donations by men in positive emotional states is explained by the lower probability of donating (Table 8.1). The phenomenon of decreasing donations after experiencing positive emotions can be seen as a compensatory behavior, as PEB can be considered as an activator of positive moods. As men tend to be more prone to moral compensation than women [67], it seems natural that men would be less generous towards the ENGO when a positive mood is highly activated. However, we observe that compensatory behaviors only occur in people experiencing positive emotions.

Compensatory behaviors are also observed in subjects in the pride treatment group who have a high level of environmental awareness. In other words, feeling pride reduces the probability that highly environmentally aware subjects will donate a positive amount to the ENGO. This would seem to indicate that individual environmental consciousness has not been internalized as a social norm [68].

To summarize, the effective monetary impact results (Tables 9.1 and 9.2) show that none of our induced emotions increase donations. On the contrary, both negative emotions (i.e., sadness and shame) reduce average donations by nearly €1.5.

## Discussion

In this paper, we investigate the impact of emotions on PEB using a laboratory experiment in which we induce emotions in subjects via hypothetical situations (script reading describing a performance situation; imagined emotions) [48]. We consider an indirect PEB, i.e., a monetary donation to an environmental non-governmental organization (ENGO) [45] as a proxy for PEB, and we measure environmental concern through the NEP scale [40].

Our results show that emotions influence subjects’ decisions to make monetary donations to ENGOs. However, the impact of a particular emotional state experienced by subjects on their willingness to donate to ENGOs is more subtle, and depends on both valence and the social dimension of emotions. This also appears to be dependent on individual characteristics such as gender and one’s level of awareness on environmental issues. Our main results are stated as follows.

Firstly, results show that only negative emotions reduce donations in comparison to donations in the control treatment, and thus only partly support Hypothesis 1. We observe behavioral consistency in subjects feeling negative emotions, and rather compensatory behaviors for subjects experiencing positive emotions. In the literature, contributions indicate that on the one hand concrete moral self-perceptions (focusing on the recent past) activate self-regulatory behaviors and then are more likely to exhibit compensatory behaviors, and on the other hand that abstract moral self-perceptions (focusing on the distant past) activate identity concerns and then are more likely to exhibit behavioral consistency [69,70]. Moreover, positive emotions appear to push people to make optimistic judgments whereas negative emotions push them to rather pessimistic ones [8,71]. As negative emotions (i.e., sadness and shame) drive people to donate less, they might adopt rational distancing and behave consistently over time, as a “way to protect oneself from painful emotions” and thus may be “less likely to engage in pro-environmental behavior” [6]. Compensatory behaviors are observed in men experiencing positive emotions (both happiness and pride), which can be explained by the combination of a higher need to compensate for men than for women [67], and compensation behaviors are also accentuated because positive emotional states drive subjects to focus on a larger time-scale [8].

Secondly, we find that social emotions impact negatively PEB unlike individual emotions which confirms Hypothesis 2. This result is mainly due to the negative impact of shame on average donations. Individual environmental awareness seems not to be sufficient to trigger a positive dynamic for the establishment of new standards in terms of donation to ENGOs.

Thirdly, we show that men donate significantly less than women do, but mainly in positive emotional states (i.e., happiness and pride), and thus Hypothesis 3-a) is partly supported. This result corroborates with findings stating that men are less likely to be eco-friendly in their attitudes, choices, and behaviors [72], and at the same time reinforces the difficulty of finding a clear-cut correlation between positive mood and selfishness [73]. Brough et al. [74] explain this gender generosity gap by the green-feminine stereotype. In other words, engaging in green behavior is considered to be “more feminine than masculine”, and therefore, men resist in adopting PEB, believing it will threaten their male identity. The green-feminine stereotype is prevalent for either gender and affects the self-esteem. Interestingly, the need to maintain a gender identity seems to be more pronounced when subjects are in positive moods.

Finally, donation levels are far higher for highly environmentally concerned subjects than for less environmentally concerned ones. However, emotions are not able to trigger PEB in either group. On the contrary, feeling pride appears to push highly environmentally concerned subjects to donate less to the ENGO. Thus, Hypothesis 3-b) cannot be confirmed.

## Conclusion and further research

Our laboratory experiment shows that triggering emotional states, especially negative ones, is counterproductive for persuading people to adopt PEB. Thus, in advertising campaigns, social media or other communication channels, relying on emotional appeals should be used with caution and needs further insights.

However, our work has several limitations.

First of all, the emotion induction was carried out by script reading. It would be interesting to investigate whether these results are confirmed under visual emotional induction (images, videos, etc.).

Second, the use of the dictator game is purely individual-subject focused. Nevertheless, real-world decision making is often deliberative and influenced by peer group behavior. It is shown that social ties and norms indeed play an important role in the enforcement of PEB and cooperation [34,35]. To go further, a focus should be made on identity issues, the link with emotions, and the way group dynamics in line with social emotions may shape norms [75]. Furthermore, other types of experimental games should be implemented such as public good games or other social dilemma games. For example, an interesting extension of this research might be to examine the role of social norms [76,77] and the way emotions may reinforce individuals to conform to norms, in order to gain insight on how to design efficient fundraising campaigns. Considering other emotional states, such as guilt or empathy, or considering other experimental games with strategic interactions, such as the public good game then, could constitute interesting extensions to analyze the impact on the establishment of social norms.

Last, another important point to investigate concerns the external validity of our results, by realizing a natural field experiment in collaboration with ENGOs. Another research path could be a focus on the emotional impact from donation decisions and the way they interact with the emotions induced by informational campaigns.

## Supporting information

S1 Fig Distribution of monetary donations to ENGOs by treatment. (TIF)

S2 Fig Distribution of monetary donations to ENGOs by treatment and by gender. (TIF)

S3 Fig Distribution of monetary donations to ENGOs by treatment and by level of environmental awareness. (TIF)

S1 Table Reported adjective following emotion induction (by treatment). (DOCX)

S1 File Scripts used to induce emotional states. (DOCX)

S2 File Laboratory experiment instructions. (DOCX)

S3 File Database. (XLSX)
