# Supplementary figures and images for: The effects of induced emotions on environmental preferences and behavior: An experimental study
Source: PLoS One. 2021 Sep 30;16(9):e0258045. doi: 10.1371/journal.pone.0258045 (PMC8483342; doi:10.1371/journal.pone.0258045)

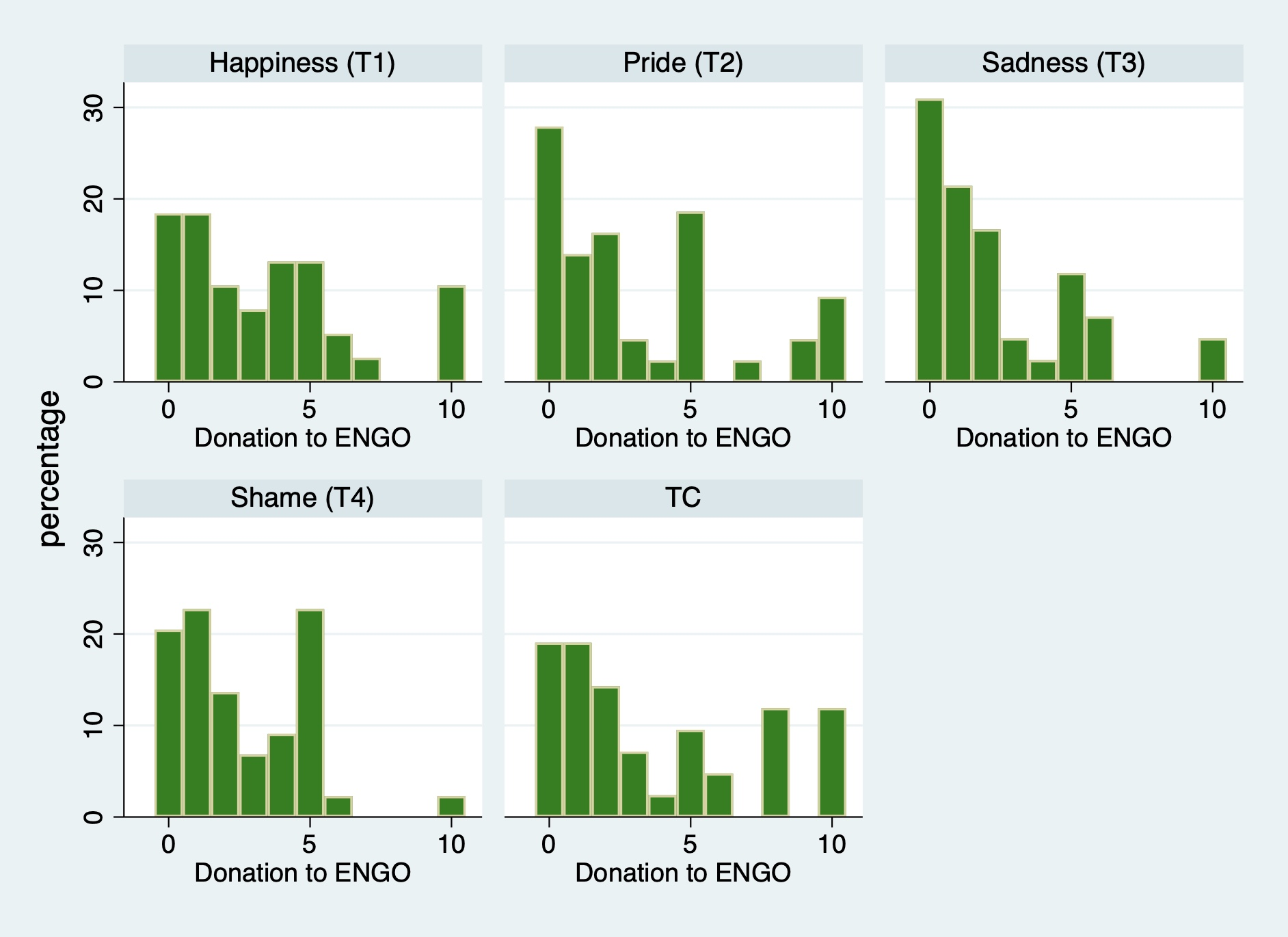

Supplement: S1 Fig — (TIF) [file pone.0258045.s001.tif]

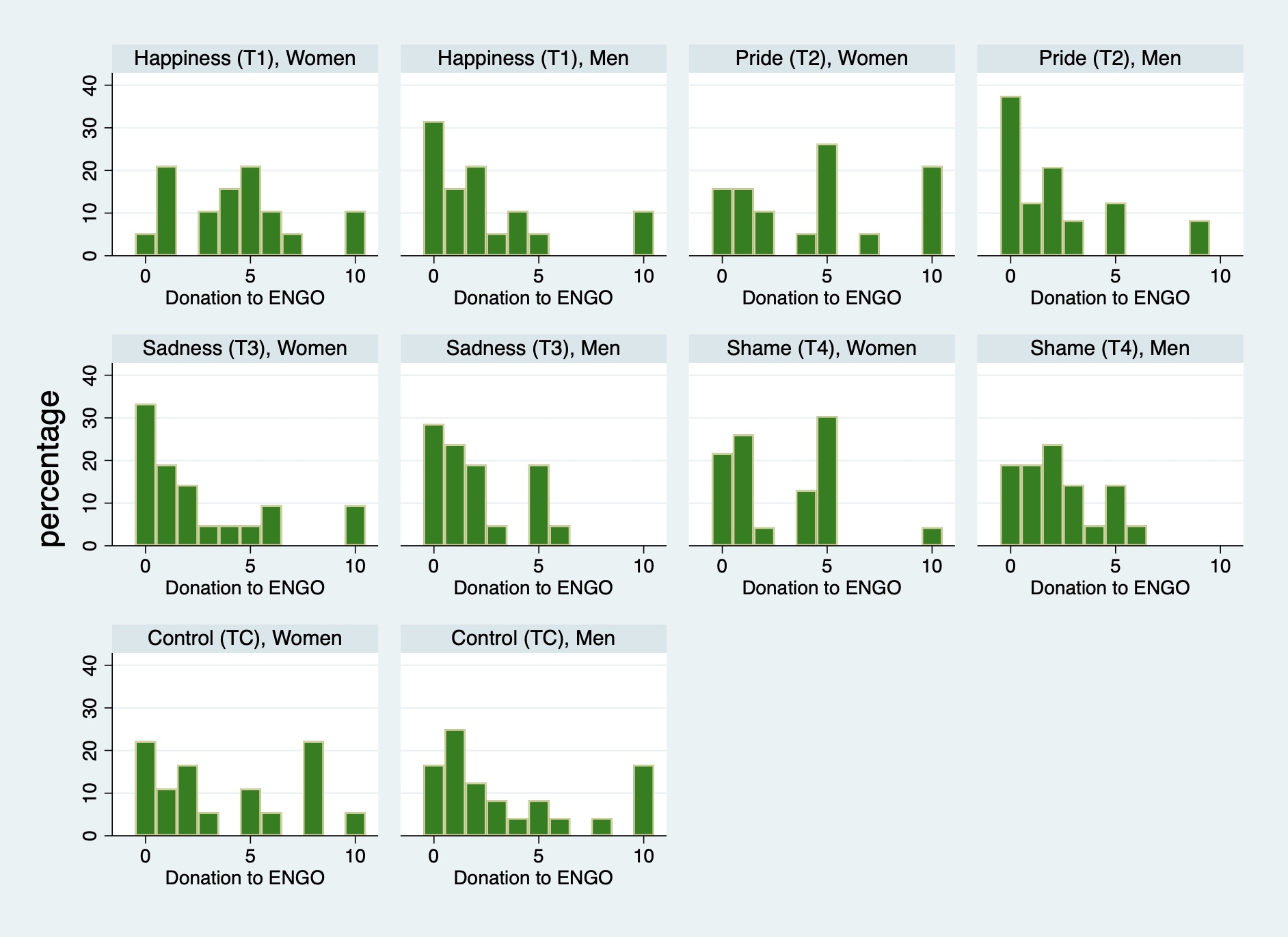

Supplement: S2 Fig — (TIF) [file pone.0258045.s002.tif]

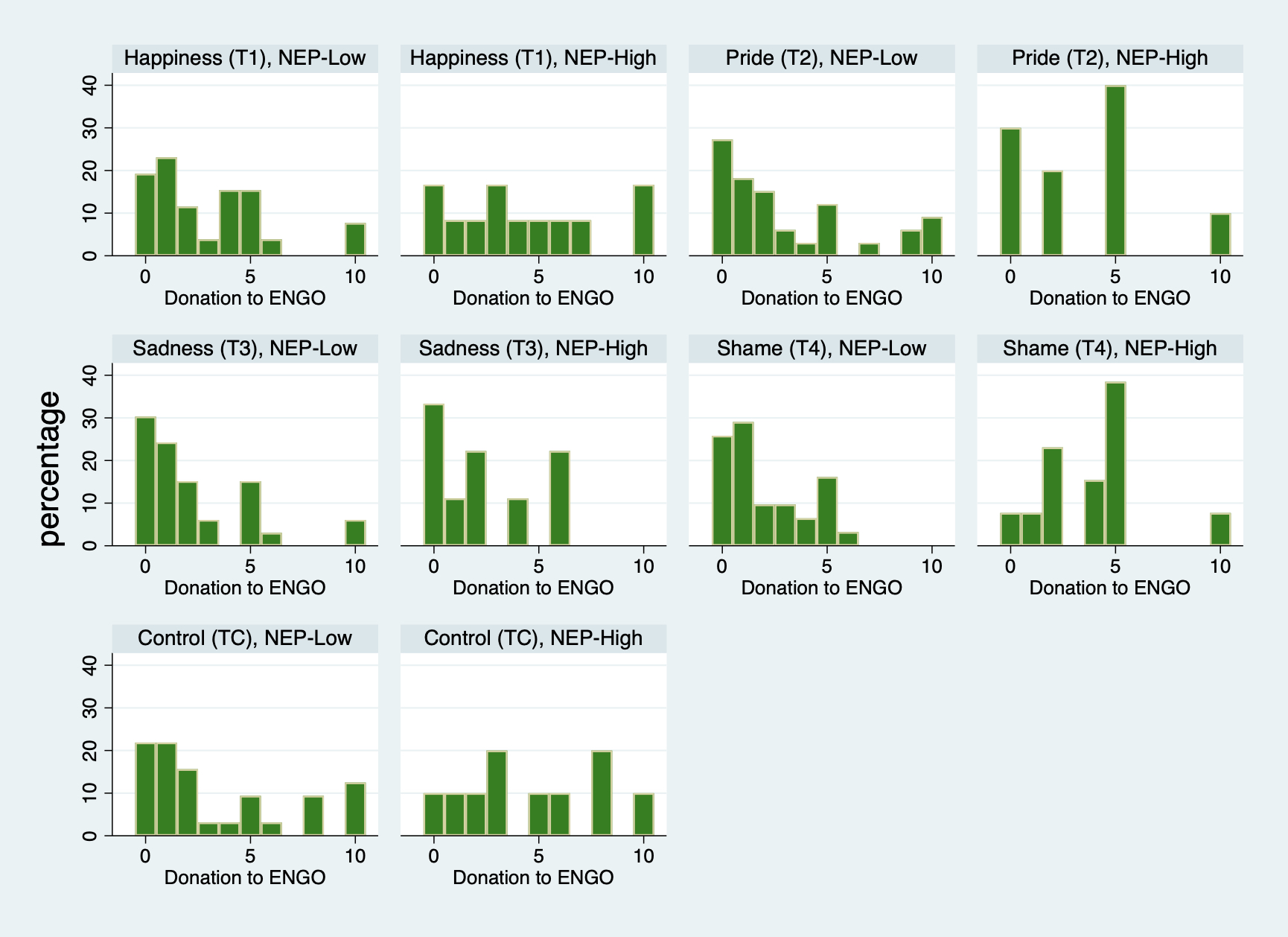

Supplement: S3 Fig — (TIF) [file pone.0258045.s003.tif]
