# Supplementary material for: The effects of induced emotions on environmental preferences and behavior: An experimental study
Source: PLoS One. 2021 Sep 30;16(9):e0258045. doi: 10.1371/journal.pone.0258045 (PMC8483342; doi:10.1371/journal.pone.0258045)
Supplement: S1 Table — (DOCX) [file pone.0258045.s004.docx]

**Table S1. Reported adjective following emotion induction (by treatment)**

|  | **Number of observations** | **Correct choice of related adjective**  **(%)** | **Partially correct choice of related adjective (valence)**  **(%)** |
| --- | --- | --- | --- |
| **Happiness (T1)** | 38 | 84.21% | 86.84% |
| **Pride (T2)** | 43 | 76.74% | 95.35% |
| **Sadness (T3)** | 42 | 52.38% | 80.95% |
| **Shame (T4)** | 44 | 65.91% | 86.36% |
| **Control (TC)** | 42 | 23.81% | - |
