# Supplementary material for: The effects of induced emotions on environmental preferences and behavior: An experimental study
Source: PLoS One. 2021 Sep 30;16(9):e0258045. doi: 10.1371/journal.pone.0258045 (PMC8483342; doi:10.1371/journal.pone.0258045)
Supplement: S1 File — (DOCX) [file pone.0258045.s005.docx]

**Supplementary material S1. Scripts used to induce emotional states**

Based on de Hooge et al. [48], we designed scripts to induce emotions for each experimental treatment (see below). Texts were tested in classes prior to the experiments. Subjects were asked to consider the following: “Imagine you are taking a course where everybody is required to give a presentation to a work group of 25 fellow students.” For each emotion, subjects read the corresponding text:

To induce happiness (T1): “Your presentation is scheduled for the day after your birthday, for which you are planning a small party, but you do not intend to cancel it. A few days before the date of your presentation, you still have not yet started preparing for it as planning your birthday party takes time. You receive a message from your teacher that informs you that the date of your presentation has been postponed by one month. As a result, you can now enjoy your birthday party to the fullest.”

To induce pride (T2): “You have prepared for your presentation carefully and designed many animated slides and illustrations (photos, videos). When you make your presentation, you're very comfortable and the students are very interested in what you have to say. They ask you lots of questions that you answer in a very skillful way. At the end of your presentation, all the students applaud you and your teacher congratulates you while inviting other students to join you for future presentations.”

To induce sadness (T3): “Your presentation is the last one of the semester. You have prepared it carefully for hours. You have designed many animated slides and illustrations (photo, video). The night before, you again go over the presentation you are going to make the next morning at 9 am. On the morning of your presentation, your alarm clock doesn’t ring and you miss your class. You know that your presentation was the last of the scheduled presentations and there is no other date. Your teacher warned you that he would not accept any excuses or postponements. So, you have done all of this work for nothing.”

To induce shame (T4): “When your turn arrives, nothing happens as expected. You don't find your words. The text of your slides is too small and is riddled with mistakes. At the end, when the teacher asks the students who have listened to you to ask questions, it becomes obvious that you are not at all familiar with your subject, and that you are unable to answer the questions. The teacher makes you feel that the presentation you have given does not meet his or her expectations and is below the level of other students.”

For the control treatment (TC): “Imagine making a presentation to 25 students. You prepared it several days in advance. When you make your presentation in front of other students, the experience is quite normal. Your presentation is at a level comparable to that of other students.”
