# Supplementary material for: The effects of induced emotions on environmental preferences and behavior: An experimental study
Source: PLoS One. 2021 Sep 30;16(9):e0258045. doi: 10.1371/journal.pone.0258045 (PMC8483342; doi:10.1371/journal.pone.0258045)
Supplement: S2 File — (DOCX) [file pone.0258045.s006.docx]

**Supplementary material S2. Laboratory experiment instructions**

*Good morning / Good afternoon*,

The experiment you are going to participate in is intended for the study of decision-making. We ask you to read the instructions carefully; they should allow you to fully understand the experiment. When you have read all of the instructions, a research assistant will read them aloud to you.

All your answers will be treated anonymously throughout the entire experiment. You will indicate your choices using the computer you are sitting in front of.

The experiment is composed of three parts, which are analyzed either by economics researchers or psychology researchers as part of their research work.

At each step, the screen will tell you whether you can complete the task or whether to wait. Waiting intervals are sometimes introduced so that everyone in the experiment can move forward at the same pace.

Your remuneration will be paid to you in cash at the end of the experiment and will be determined by your choice in Part 3.

We ask you throughout the experiment to be completely silent and not to show any signs that might influence your neighbors. Your cellphone must be turned off. You should not consult any documents other than those which have been distributed to you or which are presented to you on your computer screen.

If you have a question, raise your hand and a research assistant will come and answer you in private.

**INSTRUCTIONS FOR PART 1**

You will be associated with an environmental Non-Governmental Organization (NGO) for the entire duration of the experiment.

You have the choice between three NGOs: World Wildlife Fund (WWF); Greenpeace; or the Foundation for Nature and Man (*Fondation pour la Nature et l’Homme*).

You have at your disposal an information sheet on the NGOs to help you make your choice. There are no right or wrong answers.

**INSTRUCTIONS FOR PART 2** (distributed once Part 1 is finished)

The second part of the experiment consists of three tasks.

*Task 1:*

You will be presented with a series of questions. We ask you to respond as sincerely as possible. For each question, give your answer by moving the cursor on the gradient from 0 to 99.

For example: If you do not feel concerned, move the slider slightly to the left. On the other hand, if you feel strongly concerned, move the cursor to the right or even completely to the right.

IMPORTANT: Please answer quickly and intuitively.

*Task 2:*

A text will be presented to you. We ask you to read it in detail.

After reading, you should describe in a few lines, quickly and intuitively, what you are feeling. Please pick the adjective corresponding to your emotional state among the following ones (the adjectives were presented in a random order):

Indifferent – Happy – Delighted – Proud – Esteemed – Sad – Unhappy – Shameful – Humiliated.

You will have 5 minutes to do this.

*Task 3:*

You will be presented with a series of questions. We ask you to respond as sincerely as possible. For each question, give your answer by moving the cursor on the gradient from 0 to 99.

Same example as in Task 1.

**INSTRUCTIONS FOR PART 3** (distributed once Part 2 is finished)

The third part is made up of 2 tasks.

*Task 1:*

During this task, you are allocated an amount of €10. You are asked to make a decision regarding this €10 sum.

We give you the opportunity to donate a portion of this amount to the NGO with which you are associated (that is, the NGO that you chose in part 1 of the experiment). You can choose to donate any whole number between €0 and €10; whatever remains will be your gain.

The game will only be played once.

IMPORTANT: The researchers in charge of this experiment have committed to ensuring that all sums allocated to the environmental NGO during this experiment will actually be paid as allocated.

*Task 2:*

You will be presented with a series of questions. We ask you to respond as sincerely as possible. For each question, give your answer by moving the cursor on the gradient from 0 to 99.

Same example as in Part 2 Task 1.
